# Clinical trial site readiness for decentralized trials – fitting trials into today’s world

**DOI:** 10.1017/cts.2024.17

**Published:** 2024-02-12

**Authors:** Pamela Tenaerts, Adrian F. Hernandez, Craig Lipset

**Affiliations:** 1 Medable Inc, Palo Alto, CA, USA; 2 Duke Clinical Research Institute, Durham, NC, USA; 3 Clinical Innovation Partners, Basking Ridge, NJ, USA; 4 Decentralized Trials & Research Alliance, San Diego, CA, USA

**Keywords:** Clinical trial sites, site readiness practices, decentralized clinical trials, quality improvement, evaluation

Post-pandemic, decentralized clinical trials (DCTs) have emerged as a viable new way to efficiently conduct research. Originally piloted over 15 years ago, adoption of decentralized research methods during the COVID-19 pandemic was necessary to keep critical clinical trials running. As we emerge from the pandemic, regulators are clarifying their expectations in recommendation papers [1] and national guidance documents [2–4].

The Food and Drug Omnibus Reform Act (FDORA), signed into law in December 2022, defines a “decentralized clinical study” as a “clinical study in which some or all of the study-related activities occur at a location separate from the investigator’s location [5].” The main feature of a decentralized study is being centered around the participant and what may be most suitable for the participant’s access and experience. This may include engagement, recruitment, consent, study procedures, access to investigational product, and follow-up with a variety of data collections using active or passive methods.

Importantly, DCTs use a spectrum of methods that can be completely remote or partially decentralized with hybrid approaches (Table 1). Hybrid trials are typically defined as those that support some visits to be conducted on site, while other visits or assessments can be performed at a participant’s home or other preferred and accessible location. Fully remote trials have no required site visits, relying instead on all visits to take place using decentralized methods. Not every trial can be decentralized in the exact same way. Decentralized elements will need to be considered for every clinical trial in a fit for purpose fashion based on the condition under study, phase of the research, trial population, and procedures required in the protocol schedule of events. Any of these will determine which decentralized methods are best to be deployed. For example, complex study treatments like gene therapies in advanced stage disease trials may not be best suited for a telemedicine consent process, but might be better conducted in person [1], whereas trials for persons with decreased mobility may benefit from some trial visits conducted by telemedicine or some visits conducted by local healthcare providers. DCT methods could include but are not limited to electronic informed consent (eConsent), electronic clinical outcome assessment (eCOA), connected sensors, televisits, at-home shipments of investigational product, and other local elements such as the use of home health providers, local labs, local providers, mobile research sites, or retail pharmacies.

**Table 1.** Examples of clinical trials using decentralized elements

Trial	Initiating sector	Product(s) investigated	Indication tested	Enrollment Range	Decentralized elements used	Regulatory action	Published results (DOI)
ACTIV 6	NIH	Ivermectin, fluticasone, fluvoxamine, montelukast, metformin,	Acute COVID-19 infections (SARS-CoV-2)	1,001–5,000	fully online screening online enrollment eConsent local laboratory direct to patient shipping of study drug electronic patient-reported outcomes (ePROs) remote collection of hospitalization records	Ongoing	10.1056/NEJMoa2209421 10.1001/jama.2023.23363 10.1001/jama.2023.1650 10.1001/jama.2022.24100 10.1001/jama.2022.18590
ADAPTABLE	Academia	Aspirin	Cardiovascular disease	>10,000	online enrollment eConsent ePROs collection of patient information from electronic health records linked with Medicare and private insurance information engagement with patient partners at all levels of the study, from protocol design to creation of study materials to dissemination of results	Not submitted	10.1056/NEJMoa2102137
PEMPHIX	Industry	Rituximab	Pemphigus vulgaris	100–500	Telemedicine/ video visits via sub-study	Telemedicine patients removed from dataset used for submission	10.1056/NEJMoa2028564
Pfizer’s landmark trial	Industry	COMIRNATY (COVID-19 vaccine, mRNA)	SARS-CoV-2	>10,000	ePROs (79% of participants reported with bring your own device (BYOD) smartphone)	Approval FDA/EMA^a^	10.1056/NEJMoa2034577
REMOTE	Industry	Tolterodine	Overactive bladder	100–500	fully online screening eConsent local laboratory direct to patient shipping of study drug	discontinued	10.1016/j.cct.2014.04.009
Stop COVID	Academia	Fluvoxamine	SARS-CoV-2	100–500	Fully remote trial: fully online screening eConsent direct to patient shipping of study drug connected sensors (pulse oximeter, blood pressure monitor, and thermometer) online surveys	Not submitted	10.1001/jama.2020.22760
Stop COVID 2	Academia	Fluvoxamine	SARS-CoV-2	501–1,000	Fully remote trial: fully online screening eConsent direct to patient shipping of study drug connected sensors (pulse oximeter, blood pressure monitor, and thermometer) online surveys	Not submitted	10.1093/ofid/ofad419
WeSMA	Industry	Risdiplam	Spinal muscular atrophy	100–500	eConsent app-based questionnaires telemedicine/ video visits	Recruiting^b^	n/a

a See FDA package insert at https://www.fda.gov/media/151707/download.

b See ClinicalTrial.gov study record at https://clinicaltrials.gov/study/NCT05232929.

Historically, clinical trials have done a poor job of recruiting participants that are representative of the population living with the condition studied due to multiple factors, including access to clinical trials, distance to the trial site, and the time commitment of traveling to the trial site [6]. Decentralized methods have the potential to make trials more accessible to a larger population, make them easier to incorporate into daily life, and have the potential to collect more meaningful data on how the investigational medical product affects how trial participants feel and function. By improving trial access, decentralized trials may complement other equity initiatives and support goals for inclusion of more diverse and representative trial participants. Care has to be taken that the technology itself does not become a barrier and that digital access issues are addressed with options such as provisioned devices and paid data plans by trial sponsors. Decentralized methods can also make trials more resilient in supporting business continuity should participants be unable to reach sites for any reason, while also helping to support environmental and social responsibility objectives on the path toward more environmentally sustainable research.

As research models continue to expand the number and variety of locations to improve access, these approaches will challenge the definition of the “site” as a brick-and-mortar building and the foundational element for clinical trial readiness practices. A “building” does not conduct research—research is performed by a qualified investigator supported by people, processes, and technology surrounding a qualified investigator. Similarly, the term “investigator” may not need to be an individual but rather an organization. As we move toward more decentralized trial models leveraging the potential for systems of care or broader organizational approaches to reach people and conduct clinical trials, an organization may be more appropriate to be recognized as the “investigator.” For example, organizations routinely serve as sponsors and hold the IND with the commensurate responsibilities. Similarly, authorized officials are responsible for NIH grants or clinical care systems. So, if a system of care is being used for a clinical trial, then an investigator (the organization) is using the systems, units, personnel, and controls responsible for the trial conduct and can manage the appropriate master delegation of responsibilities. As the trial participants whom principal investigators enroll, are responsible for, and provide oversight of increase in numbers from 10s to 100s to 1000s and even more; and participation expands from local to regional to national, new models and supporting tools will need to evolve for operationalization. Select collaborative groups are working on exploring these issues and solutions, and best practices specific to oversight and responsibility should be created. Practices that promote clinical trial site readiness can be agnostic to location by modernizing our definition of a site or shifting the focus to the investigator and their supporting infrastructure.

Using the site readiness practices framework for decentralized trials requires additional considerations related to roles and responsibilities, technology access, medical licensing, and oversight (Table 2). Site readiness practices, originally described in a companion article [7], when adapted for decentralized trials provide regulators, ethics committees, and investigators and sponsors greater confidence to leverage decentralized methods to make research more accessible and more resilient. These practices help to guide investigators in the training and processes needed to support decentralized trials, while guiding study teams toward selecting the best possible investigators and sites for the needs of each study. Additionally, site readiness practices adapted for DCTs can help sites prioritize what additional skills and resources may be needed for training and other investment. Use of site readiness practices can also improve sponsor’s and CRO’s identification of appropriate sites for studies with decentralized elements.

**Table 2.** Considerations for site readiness in the context of decentralized clinical trials

Domain	Adaptation for decentralized clinical trials
Research team	■ Consider research team skills and composition, including confidence with technology and new processes, as well as supporting communications beyond in-person interactions. ■ Ensure appropriate training and mentorship regarding decentralized methods and tools (e.g., technical training on apps/platforms, participant engagement in virtual environment). ■ Plan and support the oversight and responsibility of the investigators (PI, Sub-I and Co-investigators) given diversity of data streams and expanded research team roles with tools/data/analytics and enhanced team communication. ■ Plan and account for supporting participant interactions with decentralized methods in team capacity planning. ■ Define roles for community care providers that contribute to the research team but may not be a trial or satellite site.
Infrastructure	■ Ensure sufficient access to hardware, software, and internet connectivity for research team. ■ Ensure sufficient access to hardware, software, and internet connectivity for trial participants. ■ Plan for locally maintaining and protecting digital infrastructure, including protection of participant data (e.g., avoiding repurposing of participant data without consent). ■ Separate out infrastructure for performing routine standard-of-care from infrastructure needed for decentralized trials. ■ Ensure proper budget and team capacity for decentralized approaches including support for participant interactions and oversight requirements.
Study management	■ Understand local telemedicine laws and different interpretations of investigational product distribution affecting participants across multiple jurisdictions. ■ Maintain a log of participant IT capabilities and technology needs including Wi-Fi access and required devices. ■ Understand the maintenance and documentation requirements and supporting vendors of medical devices or other equipment to be used in the trial. ■ Consider new SOPs that incorporate considerations for conducting e-Consent, e-COA, home health services, IP handling/distribution. ■ Plan for appropriate technical, clinical, and emotional support for participants engaging in remote trial activities.
Data collection and management	■ Define the role of clinical investigators in monitoring data from sensors, as well as data acquired by contracted home health providers, vendor systems, or other third parties. ■ Validate quality of data being recorded from sensors.
Quality oversight	■ Maintain and test a plan for real-time monitoring and response to potential safety issues.
Ethics and safety	■ Engage in early discussions with IRB to understand state of readiness for review of studies with decentralized methods. ■ Ensure protocols that are submitted to IRBs clearly define which components include decentralized methods. ■ Ensure appropriate level of security of patient data from breeches and hacking. ■ Incorporate privacy considerations and affirmation of participant data use into informed consent, including for trials that may use video visits, home visits, or devices that collect geolocation data. ■ Consider pharmacovigilance methods that can be applied in the context of decentralized trials. ■ Develop plan with clearly defined responsibilities for the identification and response to safety signals and adverse events.

Recent surveys have indicated sites are facing challenges with adoption of decentralized elements. A survey released by the Association of Clinical Research Professionals reveals that sites lack the training and budget to implement DCTs and that the technologies do not always take site user experience into consideration, for example, the lack of Single Sign On was mentioned as a barrier [8]. Additionally, the top-cited reason sites declined participation in decentralized trials in another site-based survey was hesitancy to adopt these methods without a sufficient budget to cover additional training or the integration of new technologies [9]. This points to the need for robust support of sites for training and adoption including the need for technology providers to take site users' experience and needs into consideration when developing new clinical trial technologies. Investigators have also raised concerns regarding oversight in decentralized research, in particular with connected devices streaming data or third parties being contracted to support some visit procedures. Regulatory recommendations and guidance have called for processes and tools to be included during study planning to address these concerns and support existing investigator obligations to safety and integrity [1,4]. Site readiness to deploy decentralized approaches needs to be ensured with specific training programs, intuitive easy to use software and applications, and sufficient budgets. The site readiness practices and the related adaptations for DCTs should help alleviate some of the challenges encountered by sites.
